# Life-threating outcomes after dental implantation in patient with idiopathic thrombocytopenic purpura: a case report and review of literature

**DOI:** 10.1186/s40902-018-0178-9

**Published:** 2018-12-10

**Authors:** Sung-Tak Lee, Jin-Wook Kim, Tae-Geon Kwon

**Affiliations:** Department of Oral and Maxillofacial Surgery, School of Dentistry, Kyungpook National University, 2177 Dalgubeol-daero, Jung-gu, Daegu, 41940 Republic of Korea

**Keywords:** Thrombocytopenia, Idiopathic, Dental implant, Bleeding

## Abstract

**Background:**

Patients with chronic ITP (idiopathic thrombocytopenia) frequently do not require comprehensive medication for daily life. Usually, it had been regarded that postoperative bleeding after a simple or surgical extraction is easily controlled by simple local measures even in patients with ITP. This lack of regular medication usage can sometimes lead practitioners or patients to underestimate the potential life-threatening risk of ITP. There had been no report on postoperative hemorrhage in a patient with ITP related to dental implant surgery.

**Case presentation:**

This report presented a life-threatening postoperative hemorrhage after dental implant surgery in an adult with chronic ITP and subsequent emergency management after severe bleeding and airway compromise.

**Conclusion:**

The presented case emphasizes the thorough hematological evaluation of the patients even for patients who do not take any specific medications for asymptomatic, chronic ITP.

## Background

Idiopathic thrombocytopenic purpura (ITP) is a relatively common and easily recognizable acquired autoimmune disorder that presents with a low platelet count. It is characterized by the immune-mediated destruction of platelets by autoantibodies and the inhibition of platelet release from megakaryocytes. A normal platelet count ranges from 150,000 to 450,000/μL, and lower counts are typically referred to as thrombocytopenia. Low platelet counts often lead to severe postoperative bleeding, spontaneous mucosal bleeding, cutaneous ecchymosis, retinal hemorrhage, and even life-threatening bleeding [1]. The ITP guideline panel of the American Society of Hematology estimated that patients with platelet levels below 30,000/μL have an approximately 5% risk of fatal hemorrhage over their lifetime [2]. However, considering the large variations in bleeding risk among different age groups, the risk of fatal hemorrhage is likely to be even higher than this estimate [3].

Despite the potential risk, many patients with ITP need only close medical observation without special treatment. Because spontaneous bleeding is rare unless the number of platelets is < 30,000/μL, therapeutic intervention is typically performed only when the platelet count falls below this level [4]. Therefore, a surgical procedure is not necessarily safe for a patient who is not being treated with a transfusion or medication. Before performing a surgical procedure, it is necessary to identify the family and medical histories, medication status, and detailed history of any bleeding tendency. Postoperative bleeding after oral surgery occurs in 8.6–32.1% of patients with compromised hemostasis [5].

Postoperative bleeding has been reported in patients with ITP after minor oral surgical procedures such as crown lengthening, cyst enucleation, and tooth extraction [6, 7]. The risk of a fatal bleeding event in a patient with a low platelet count is reportedly relatively high. Moreover, patients with ITP and persistent low platelet counts have a poor prognosis [8]. However, a previous study suggested that postoperative bleeding after a simple or surgical extraction is easily controlled by simple local measures in patients with ITP [9].

No study has been conducted on postoperative hemorrhage in a patient with ITP related to dental implant surgery. The present case report describes a life-threatening outcome after implant surgery in a patient with ITP and reviews the adequate management thereof.

## Case presentation

A 55-year-old man with a past history of ITP and hypertension visited a local dental clinic. Four years earlier, the patient had undergone knee joint surgery and was diagnosed with ITP. Because his platelet count was maintained at 35,000/μL thereafter and no spontaneous bleeding occurred, the patient attended periodic follow-up visits to check his platelet level but was not treated with any medication. The patient underwent extraction of the left mandibular first molar because of chronic periodontitis. Post-extraction bleeding occurred for 1 week after the procedure, but the hemorrhage gradually decreased and was finally controlled by a suture at the extraction site. After the extraction, the patient’s platelet count was evaluated and maintained at 35,000/μL.

**Fig. 1:**
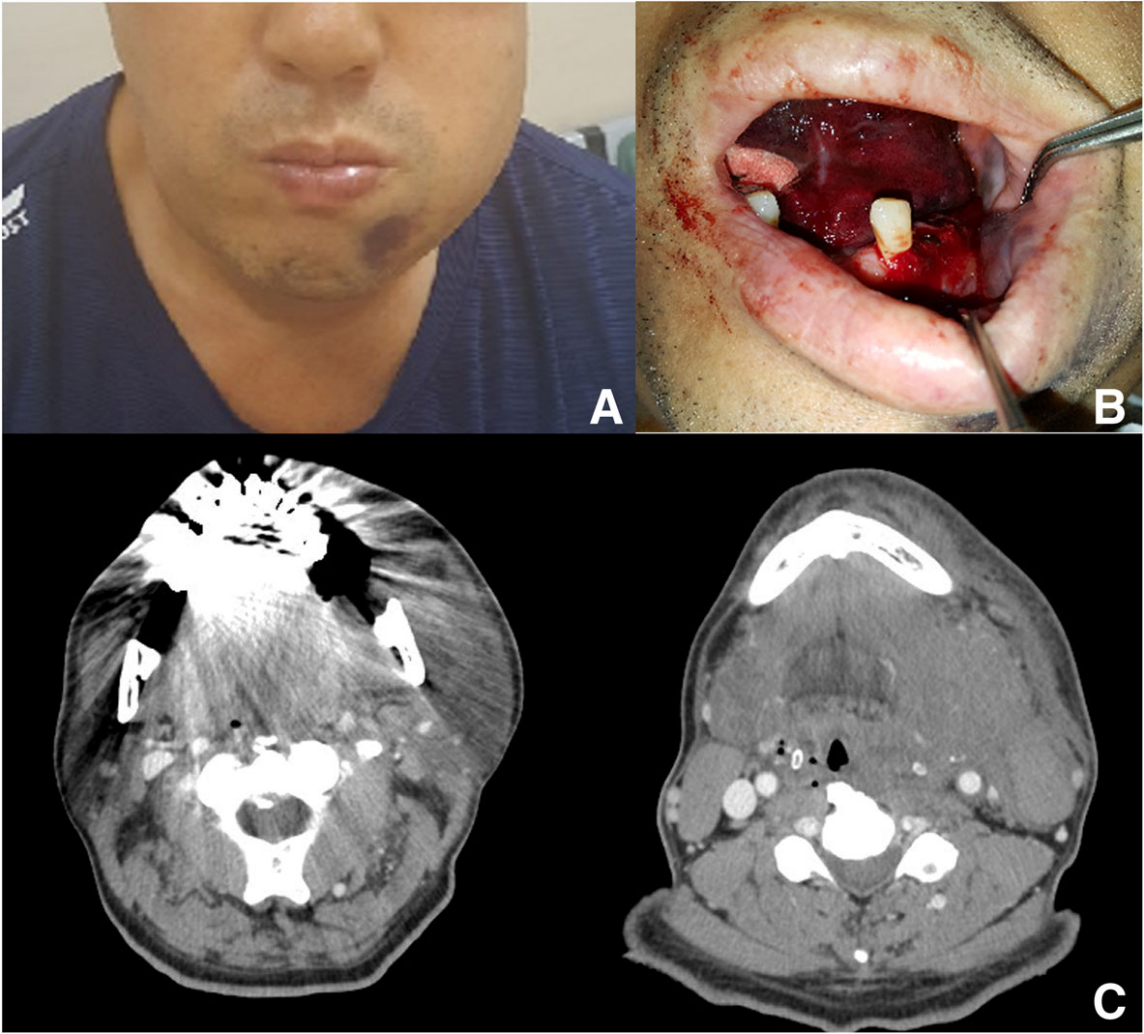
**a** Facial swelling of an emergency room arrival. **b** Intra-oral view: mouth floor elevation and bleeding at the implantation site. **c** CT axial view showed severe narrowing pharyngeal airway due to soft tissue swelling in the left masticatory, mandibular spaces, hypopharynx

One month after the extraction, the patient underwent dental implant surgery at the same site (the left mandibular first molar) at the same local dental clinic. Bleeding started immediately after fixture installation and the mouth floor began to swell. Therefore, the patient visited the emergency room (ER) of the authors’ hospital. The patient was prescribed medication to control hypertension but no medication for ITP. The patient complained of severe swelling on the mouth floor and shortness of breath accompanied by marked dysphagia, which had also occurred immediately after the surgery. Bilateral submandibular swelling and mouth-closing difficulty were present (Fig. 1).

Regarding vital signs, his blood pressure was 173/108 mmHg and the respiratory rate was 22/min. A laboratory blood workup revealed a white blood cell count of 4.38 × 10^3^/μL, a hemoglobin (Hb) level of 11.7 g/dL, and an initial platelet count of 22,000/μL. Coagulation studies revealed that fibrinogen levels (114 mg/dL) were lower than normal, and D-dimer levels were elevated at 31.91 μg/mL. Prothrombin time (PT) and activated partial thromboplastin time (aPTT) were within normal limits. A diagnosis of exclusion for ITP was made by the hematologist after ruling out other conditions that may cause symptoms similar to those of thrombocytopenic purpura. Because the patient had no past history of other contributing medication or medical condition and the results of laboratory tests showed a low platelet count and normal PTT and aPTT values, he was diagnosed with refractory ITP.

Facial-enhanced computed tomography revealed severe narrowing of the pharyngeal airway space. Marked soft tissue swelling was observed with isodensity in the left masticatory and submandibular spaces and hypopharynx. The patient complained of worsening respiration in the ER. The expanding hemorrhage was deemed to potentially result in a compromised airway with a life-threatening outcome. Therefore, the surgical wound from the dental implant was opened to evacuate the hematoma and an emergency tracheostomy was performed under local anesthesia and intravenous sedation to maintain airway patency. The patient was then admitted to the intensive care unit (ICU).

After tracheostomy, the intubation site also bled heavily. The patient was treated with antibiotics, high-dose steroids, and intravenous (IV) immunoglobulin O with a platelet infusion (platelet concentration 11 pints, 3600 mL) continuously for 3 days. The aim was to elevate the platelet level to 80,000/μL. To compensate for the blood loss (Hb 8.7) associated with constant severe bleeding from a gingival surgical wound and tracheostomy site, a transfusion of filtered red blood cells and IV saline was performed.

**Fig. 2:**
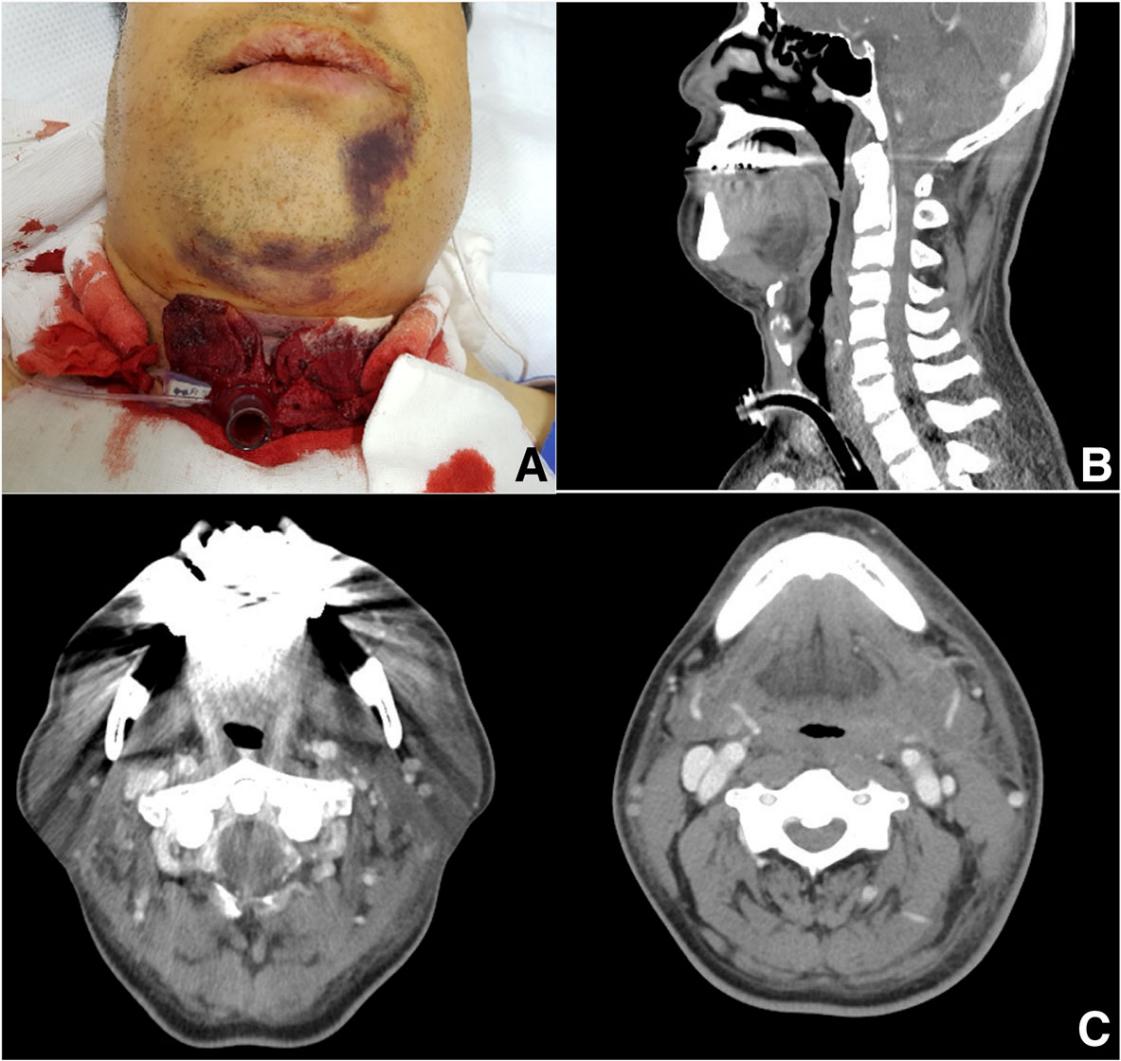
**a** Constant bleeding at the tracheostomy site after an emergency tracheostomy. **b** Follow-up sagittal CT view and **c** axial CT views after 3 days showed decreased soft tissue swelling along the superficial and deep neck spaces at 3 days after tracheostomy

Laboratory data after 3 days in the ICU showed a platelet count of 129,000/μL and other complete blood count (CBC) values within normal ranges. Follow-up CT 3 days later revealed that the hemorrhage had not expanded further. Moreover, diminished soft tissue swelling was noted along the superficial and deep neck spaces compared with the swelling shown on the previous CT images (Fig. 2). Therefore, the patient was extubated without dyspnea. The patient showed resolution of the mouth floor swelling, and no additional bleeding was detected; thus, he was discharged 9 days after admission.

**Fig. 3:**
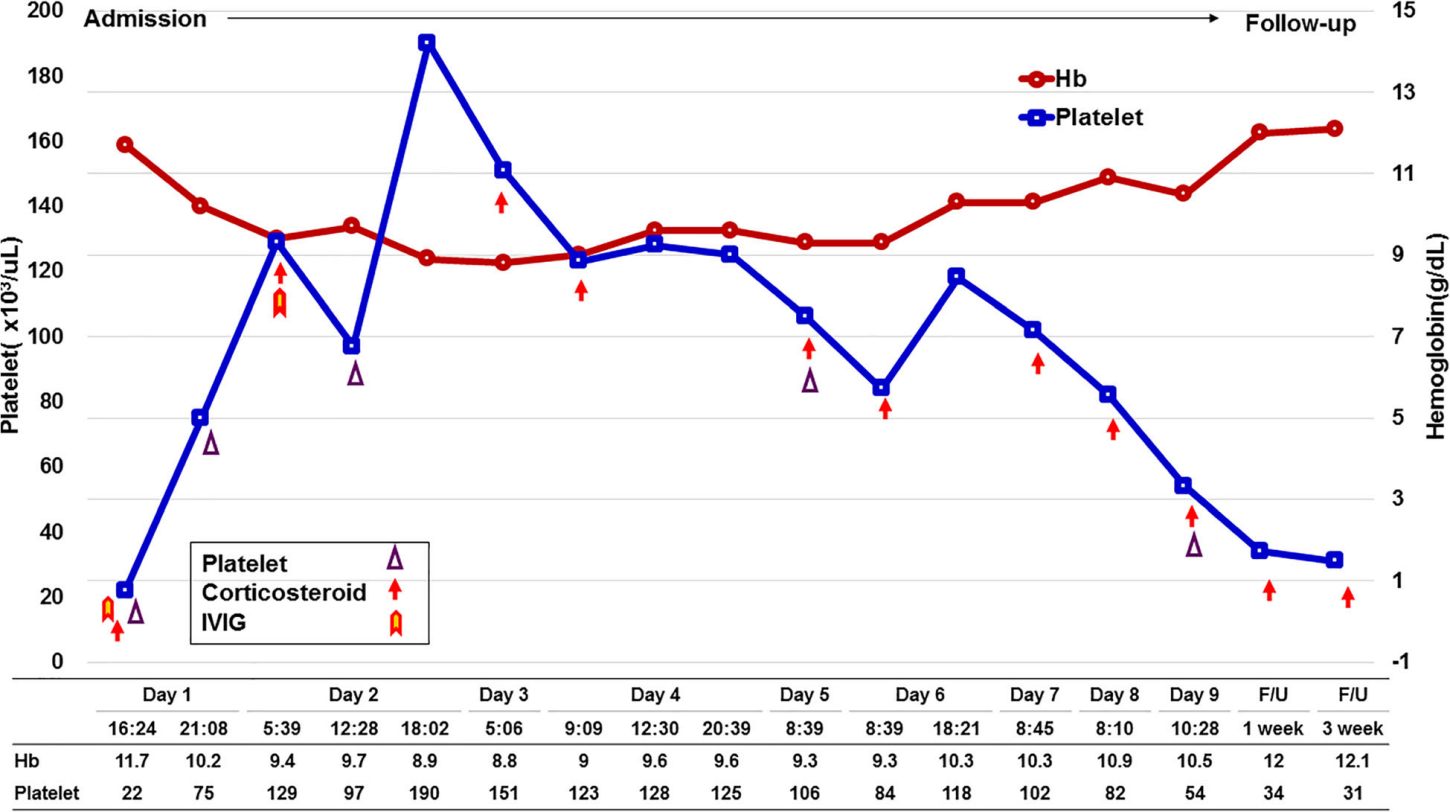
Daily change of platelet count and hemoglobin. Platelet count (× 10^3^/uL); Hb hemoglobin (g/dL); PLT (ml), platelet transfusion with platelet concentrate and plateletpheresis (ml); corticosteroid, (admission: solumedrol, mg, IV, discharge: solondo, mg); IVIG, intravenous immunoglobulin (IV-globulin SN, g, IV)

A follow-up visit 1 week after discharge revealed complete resolution of intra-oral and extra-oral swelling without superficial or deep neck infection. However, despite medication for ITP, the patient’s platelet count decreased after 1 and 3 weeks (Table 1, Fig. 3). Four months later, final prosthetic implant restoration was performed in the prosthetic department.

**Table 1:** Laboratory value of the patient

	Admission															Follow-up	
	1 day 16:24	1 day 21:08	2 day 05:39	2 day 12:28	2 day 18:02	3 day 05:06	4 day 09:09	4 day 12:30	4 day 20:39	5 day 08:39	6 day 08:39	6 day 18:21	7 day 08:45	8 day 08:10	9 day 10:28	1 week	3 week
F-RBC			250	250	250												
PLT	2000	1600															
A-PLT				640							180				289		
FFP			340														
Steroid	62.5		62.5			62.5	62.5			62.5	62.5		62.5	62.5	62.5	60	40
IVIG	80		80														

*F-RBC* filterd RBC (ml), *PLT* platelet concentrate (ml), *A-PLT* plateletpheresis (ml), *FFP* fresh frozen plasma (ml), steroid; corticosteroid (admission: solumedrol, mg, IV, discharge: solondo, mg), *IVIG* intravenous immunoglobulin (IV-globulin SN, g, IV)

## Discussion

**Fig. 4:**
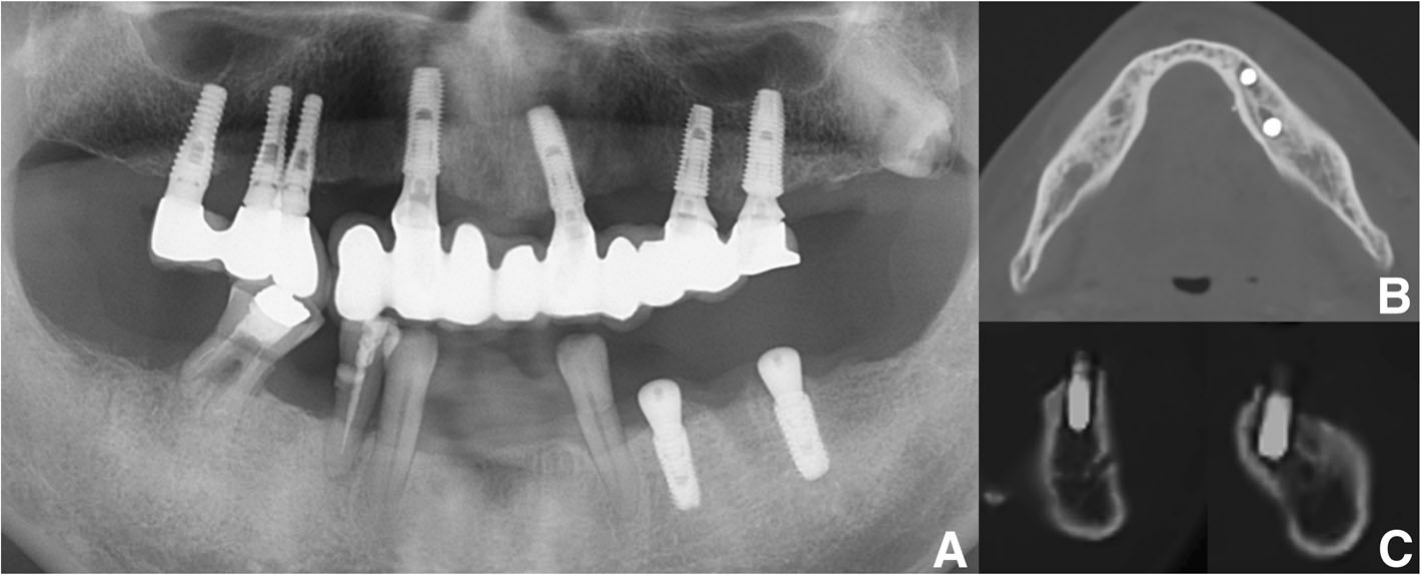
**a** Panoramic view: dental implant installation state on mandible Lt. Posterior area (bleeding area). **b** Axial CT view and **c** sagittal CT view on implant site showed adequate placement of dental implant without lingual cortex perforation and damage of adjacent anatomic structure. The dental implant installation in the left posterior mandibular area

Most reports on oral bleeding associated with ITP describe preoperative management and management of spontaneous gingival oozing and bruising as oral symptoms of ITP [8, 10]. Furthermore, only two reports have described bleeding after surgery or procedures in patients with ITP [1, 11] (Table 2), and these studies did not cover life-threatening massive bleeding after tooth extraction or dento-alveolar fracture, as in the present report. In addition, most published studies on severe postoperative hemorrhage after dental implant surgery mention that the majority of life-threatening hemorrhages result from inadequate surgical skill and damage to an anatomical structure, such as rupture of the lingual artery or mandibular lingual cortex perforation, which typically occurs in the anterior region including the premolars between the mental foramens [12–14] (Fig. 4). The dental implant in this case report was placed in the posterior mandibular area, which had no atrophic status, and no anatomic structural damage or lingual cortex perforation had occurred.

**Table 2:** Reported cases of oral hemorrhage after surgery or procedure in oral and maxillofacial area

Reference	Age/sex	History of ITP	Other PMH	Related surgery or procedure	Platelet count before treatment	Management	Platelet count after treatment
Finucane et al. (2003) [9]	13/M	Known, No med.	N/S	Tooth luxation Partial alveolar bone fracture	15,000	IVIG	70,000
Martini et al. (2011) [8]	77/F	Newly, No med	HTN, DM	Tooth extraction	20,000	PLT concentrate	87,000

*ITP* idiopathic thrombocytopenia, *HTN* hypertension, *DM* diabetes mellitus, *IVIG* intravenous immunoglobulin, *PLT* platelet transfusion

The present report shows a life-threatening hemorrhage after dental implant surgery in a patient with ITP, which is an autoimmune disorder, characterized by both immune-mediated platelet destruction and suppressed platelet production in the absence of other causes of thrombocytopenia. The role of platelets as a component of primary hemostasis is highly critical and starts the cascade of thrombus formation. Blood platelet levels are normally within the broad range of 150–400 × 10^3^/μL. Therefore, decreased production of platelets, increased platelet destruction and consumption, abnormal platelet distribution, and dilution or loss of platelets can induce congenital thrombocytopenia, an impaired bleeding tendency, and other primary hemostasis abnormalities. Therefore, ITP is a potentially life-threatening disease because of the tendency for massive bleeding and spontaneous hemorrhage as a serious complication; intracranial hemorrhage is the most frequent cause of death [15, 16].

ITP is diagnosed by exclusion. With no history of drugs or other comorbidities related to coagulopathy, a decrease in the platelet count on a CBC and a peripheral blood smear with a normal appearance should be observed. Consequently, bleeding time is prolonged. By contrast, PTT and aPTT are normal because the coagulant factor is normal. Moreover, bone marrow studies may reveal normal-to-increased levels of megakaryocytes varying from normal to immature [17].

Dental treatment is very closely related to bleeding. A bleeding tendency is critical in daily dental practice, particularly during oral surgery. Therefore, the evaluation of a patient’s bleeding risk at the initial visit is crucial. Dentists must be aware that ITP is among the diseases related to an increased bleeding tendency because of a low platelet count. Although various hemostatic materials and specialized surgical techniques are used to prevent bleeding, their utility is limited in patients with ITP undergoing dental extraction because of their thrombocytopenic condition [9]. Therefore, both dentists and hematologists must be involved in treatment planning to avoid a life-threatening complication. They must discuss possible complications and perioperative management to ensure safe dental care [18]. In general, patients with platelet counts of at least 30 × 10^3^/μL require no treatment unless a procedure inducing blood loss is required, such as a dental extraction [10], which can result in major bleeding complications in patients with platelet counts lower than 100 × 10^3^/μL [19]. Some guidelines for the management of ITP in adults suggest the following recommendations for safe platelet counts: 10 × 10^3^/μL for routine dentistry, 30 × 10^3^/μL for tooth extraction, 30 × 10^3^/μL for a regional dental block, 50 × 10^3^/μL for minor surgery, and 80 × 10^3^/μL for major surgery [20]. Other guidelines for the treatment of ITP suggest similar recommendations, as follows: platelet counts of 20–30 × 10^3^/μL for simple dental prophylaxis, 43 × 10^3^/μL for simple dental extractions, and 45 × 10^3^/μL for more complex procedures [19].

A standard plan for adult patients with a platelet count of at least 30 × 10^3^/μL is monitoring only, without intervention. However, when platelet counts decrease below 30 × 10^3^/μL, oral corticosteroids must be administered. If a rapid increase in platelet counts is required because of life-threatening bleeding, such as an intracranial hemorrhage or severe organ bleeding, as in the present study, the administration of IV immunoglobulin and/or high-dose corticosteroids is recommended [21]. In an emergency situation such as that observed in our case, platelet transfusions are also necessary [22]. The patient’s platelet count was 129 × 10^3^/μL after 3 days in the ICU with platelet transfusion and high-dose corticosteroid and IV immunoglobulin administration. However, despite treatment, his platelet count decreased to 30 × 10^3^/μL after 1 month. Such patients unable to maintain a safe platelet count with first-line treatment are defined as having refractory ITP. Excessively low platelet count resulting from refractory ITP is a strong contraindication for any surgical procedure [23].

According to the past history of the patient in this case, tooth extraction was performed 1 month before dental implantation and no abnormal bleeding events occurred. Additionally, the patient took no medication for ITP for several years and only visited his hematologist annually. The dentist at the local dental clinic may have considered the ITP to be stable because the patient was not taking medication such as for hypertension or diabetes. Consequently, dental implant surgery was considered safe because no bleeding events had occurred during a previous tooth extraction and maxillary dental implant surgery performed several years previously. The absence of relevant medication history, such as anticoagulants, does not necessarily indicate that a patient can safely undergo an operation. The dentist was unfamiliar with ITP; nevertheless, a preoperative evaluation should have been performed, including a CBC and tests for platelet count, PTT, international normalized ratio (INR), and a PTT [24]. Many dentists underestimate the bleeding risk of procedures. Moreover, some dentists often feel inadequately trained to manage these patients in the event of any bleeding complication. However, paying attention to basic procedures such as history taking, physical examination, and related laboratory blood tests can prevent events such as excessive bleeding.

Most patients with ITP and other hematologic diseases can be managed safely during dental procedures if dentists take the nature and severity of these diseases into consideration. Exact medical history taking is necessary for all patients who will undergo surgical treatment. Vital signs should be measured and the need for additional preoperative tests and their type should be determined. Practitioners must also involve a hematologist for the evaluation of the medical condition and treatment planning of each patient.

Patients with chronic ITP frequently do not require comprehensive medication for daily life, and this lack of regular medication usage can sometimes lead practitioners or patients to underestimate the potential life-threatening risk of ITP. This report presented the emergency management of a life-threatening postoperative hemorrhage after dental implant surgery in an adult with chronic ITP. However, such events are preventable with adequate perioperative evaluation and management for ITP.

## Conclusion

For a safe and satisfactory outcome for implantation in ITP patients, we recommend (1) obtaining a thorough past medical history with adequate preoperative laboratory tests such as CBC, platelet count, bleeding time, PT, INR, and aPTT; (2) not relying on the dentist’s own decision when evaluating a patient’s medical status; (3) multidisciplinary involvement among dentists, hematologists, and other related physicians; and (4) perioperative platelet transfusion and corticosteroid and IV immunoglobulin administration to achieve suitable platelet count and function when a surgical procedure is needed.

## Abbreviation

ITP: Idiopathic thrombocytopenic purpura
